# Contact-independent exposure to *Rhodococcus rhodochrous* DAP96253 volatiles does not improve the survival rate of *Myotis lucifugus* (little brown bats) affected by White-nose Syndrome

**DOI:** 10.7717/peerj.15782

**Published:** 2023-10-18

**Authors:** Sarah Hooper, Sybill Amelon

**Affiliations:** 1 Department of Veterinary Pathobiology, University of Missouri - Columbia, Columbia, MO, United States of America; 2 Department of Biomedical Sciences, Ross University School of Veterinary Medicine, Basseterre, Saint Kitts and Nevis; 3 USDA US Forest Service Northern Research Station, Columbia, MO, United States of America

**Keywords:** White-nose Syndrome, *Pseudogymnoascus destructans*, Little brown bats, *Myotis lucifugus*, *In situ* treatment trial, Bats, *Rhodococcus rhodochrous*, Hibernation, Wildlife disease, Conservation medicine

## Abstract

Since the emergence of White-nose Syndrome, a fungal disease in bats, caused by *Pseudogymnoascus destructans,* hibernating populations of little brown bats (*Myotis lucifugus*) have declined by 70–90% within *P. destructans* positive hibernacula. To reduce the impact of White-nose Syndrome to North American little brown bat populations we evaluated if exposure to volatile organic compounds produced by induced cells from *Rhodococcus rhodochrous* strain DAP96253 could improve the overwinter survival of bats infected by *P. destructans*. Two simultaneous field treatment trials were conducted at natural hibernacula located in Rockcastle and Breckinridge counties, Kentucky, USA. A combined total of 120 little brown bats were randomly divided into control groups (*n* = 60) which were not exposed to volatile organic compounds and treatment groups (*n* = 60) which were exposed to volatile organic compounds produced by non-growth, fermented cell paste composed of *R. rhodochrous* strain DAP96253 cells. Cox proportional hazard models revealed a significant decreased survival at the Rockcastle field trial site but not the Breckinridge field site. At the Breckinridge hibernacula, overwinter survival for both treatment and control groups were 60%. At the Rockcastle hibernacula, Kaplan-Meier survival curves indicated significantly increased overwinter survival of bats in the control group (43% survived) compared to the treatment group (20% survived). Although complete inhibition of *P. destructans* by volatile organic compounds produced by induced *R. rhodochrous* strain DAP96253 cells was observed *in vitro* studies, our results suggest that these volatile organic compounds do not inhibit *P. destructans in situ* and may promote *P. destructans* growth.

## Introduction

Emerging fungal pathogens are inducing disease-driven species extinction events worldwide, leading to biodiversity loss (Fisher, Gow & Gurr, 2016; Fisher et al., 2012; Smith, Sax & Lafferty, 2006). One such fungal pathogen, *Pseudogymnoascus destructans*, was first documented in Howes Cave near Albany New York in 2006 (Blehert et al., 2009). Due to the visible white fungal growth on the muzzles, ears, and/or wing membranes on some hibernating bat species, bats with active *P. destructans* infection are deemed to have the disease, White-nose Syndrome (WNS) (Chaturvedi et al., 2010).

Since that initial detection, *P. destructans* has spread to 43 US states and 10 Canadian provinces (US Fish and Wildlife Service, 2022a). As of Summer 2022, it 12 bat species are recognized to have the potential to become ill from WNS (US Fish and Wildlife Service, 2022b),with a range of clinical and behavioral signs observed. The “white fuzz” on the wings and muzzle is considered the classical clinical sign (Gargas et al., 2009); however, additional clinical signs of WNS affected bats may include dehydration and electrolyte imbalances due to epidermal erosions (Cryan et al., 2010; Verant et al., 2014) and depleted fat reserves due to shorter torpor duration (Reeder et al., 2012). Observed behavioral changes may include foraging during daytime hours (Boyles, Dunbar & Whitaker, 2006) or changing their roosting behaviors by hibernating in the colder portions of hibernacula near the front entrances (Loeb & Winters, 2022) or clustering species may roost individually (Wilcox et al., 2014).

In 2022, the US Fish and Wildlife Service (USFWS) proposed to reclassify the northern long-eared bat (*Myotis septentrionalis*) from threatened to endangered status due to severe declines and regional extirpation from hibernacula within three years of WNS detection (Frick et al., 2015; Reynolds et al., 2016). The population of three additional hibernating species, little brown bats (*Myotis lucifugus*), Indiana bats (*M. sodalis*), and tricolored bats (*Perimyotis subflavus*), have been recognized to have suffered the most severe declines with 70–90% mortality observed at infected hibernacula (Frick et al., 2015; Langwig et al., 2017). This high mortality has led to WNS being classified as an extreme threat to these three species based upon NatureServe criteria (Cheng et al., 2021).

With such precipitous population declines, a number of *in vitro* studies have been conducted to assess potential treatments. Many of these studies have yielded potentially promising compounds including topical treatments such as cold-pressed, terpeneless Valencia orange oil (Boire et al., 2016) and extracts of fungal metabolites (Rusman et al., 2020), probiotics such as *Pseudomonas fluorescens* aimed to alter the skin microflora to decrease disease susceptibility (Hoyt et al., 2015a), and exposure to volatile organic compounds (VOCs) produced by bacteria such as *Rhodococcus rhodochrous* DAP 96253 (RRDAP) (Cornelison et al., 2014) or by pure chemical compounds such as mushroom alcohol (1-octen-3-ol) (Padhi et al., 2018). Few of these potential treatments have undergone *in vivo* studies or progressed to field treatment trials (Hoyt et al., 2019).

Due to the challenges of treating large populations of hibernating bats with topical products, we selected to pursue VOC based treatments and conducted two small *in vivo* laboratory trials with RRDAP VOCs. The first *ex situ* study exposed healthy, hibernating little brown bats in hibernation chambers to RRDAP VOCs for 72 h (Pierce et al., 2017). Without any signs of toxicity or behavioral changes after 30 days post-exposure, a small second ex situ trial was conducted with little brown bats clinically affected with WNS (Pierce et al., 2017). The RRDAP exposure group was exposed for 24 h to VOCS. After 71 days, 60% of the RRDAP VOC exposure group survived whereas 0% survived in the control group (Pierce et al., 2017). Based upon these ex *situ* studies, we hypothesized that exposure to RRDAP VOCs would increase the overwinter survival of little brown bats in a field setting (*in situ*). Therefore, the goal of this study was to determine if overwinter survival of little brown bats could be increased by exposure RRDAP VOCs at two hibernacula within the state of Kentucky.

## Materials & Methods

### Ethics statement

Capture, handling, and sample collection protocols for the field treatment trial were conducted under an approved University of Missouri Institutional Animal Care and Use Committee (IACUC) protocol (#8551). Due to the high energetic costs associated with endothermic arousal from torpor (Currie, Noy & Geiser, 2015), constant disturbance would result in the mortality of all bats within the trial; therefore, the IACUC approved visual assessments of the bats during routine hibernation surveys. Authorized state biologists employed by the Kentucky Department of Fish and Wildlife were permitted (KDFWR-W-2014-01) for all described collection, treatment, and sampling procedures.

### Field sites

State biologists assisted with the selection of two known little brown bat cave hibernacula sites with one site located in Rockcastle county and one in Breckinridge county, Kentucky, USA. The hibernacula in Rockcastle county is a limestone cave approximately 2 miles long. In the winter months, the cave is used primarily by a large population Indian bats and smaller populations of little brown bats and tricolored bats. This cave was confirmed positive for *P. destructans* and bats were observed with clinical signs of White-nose Syndrome (*i.e.,* white fungal growth on nose) two years prior to the start of the study (US Fish and Wildlife Service, 2022b). The hibernacula in Breckinridge county is a limestone cave approximately 1 mile long and used by Indiana bats, little brown bats, tricolored bats, and big brown bats(*Eptesicus fuscus*). This cave was confirmed positive for *P. destructans* and bats were observed with clinical signs of White-nose Syndrome three years prior to the start of the study (US Fish and Wildlife Service, 2022b). Both caves are surveyed annually, and the dates selected for checking on the bats were selected to coincide the approximate date of the previous year’s surveys. Therefore, we were unable to survey each site on the exact same days post-treatment.

### Placement of enclosures

We suspended Nylon mesh enclosures (116 cm × 76.2 cm × 40.64 cm; Apogee Reptarium, Dallas, TX, USA) within a PVC frame constructed with 3.81 cm diameter PVC pipe and covered with a 12-guage wire mesh (2.54 × 2.54 cm) to protect the nylon enclosure from potential predators. Enclosures were bolted to the ceiling of the two field sites within the rooms where bats were found hibernating (Appendix S1). Enclosures were equipped with 650 mL reservoir reptile water bottles (Zoo Med Repti Rock Reservoir™, San Luis Obispo, CA, USA) to allow bats to express normal behavior.

### Collection and screening of bats for *P. destructans*

Hibernating bats were collected after approximately one month of hibernation. At each field site, bats were collected by hand and placed into individual sterile cloth bags. After collection, each bat had a uniquely numbered, ear sticker (Fastsigns International Inc, Carrolton, TX, USA) placed on the pinna, and evaluated morphometrically for mass (Acculab Pocket Pro 150-B; Acculab, Edgewood, NY) and forearm length.Photos of each bat’s wings were taken with illumination/transillumination of wing membranes with long-wavelength UV light (360–385 nm) to detect fungal erosion fluorescence indicative of *P. destruction* infection following the methods previously described by the authors (Amelon, Hooper & Womack, 2017). Bats were categorized as having high, medium, or low infection based upon the estimated percentage of the patagia where fluorescence was detected (>60%, 40–60%, <40%, respectively) to account for fungal load. Only those with fluorescence indicative of active infection, fungal hyphae penetrating the epidermis into the dermis, as previously described by Turner et al. (2014) were enrolled in the trial.

### Treatment with *R. rhodochrous* DAP 96253

At the Breckinridge county hibernacula, 40 bats observed to have the characteristic fluorescent orange-yellow color fluorescence of *P. destructans* infection on the patagium were randomly assigned to the control group (*n* = 20, 19 males, one female) and the treatment group (*n* = 20, 19 males, one female). At the Rockcastle county hibernacula, 80 bats observed to have the characteristic fluorescent orange-yellow color fluorescence of *P. destructans* infection on the patagium were randomly assigned to the control group (*n* = 40, 26 males, 14 female) and the treatment group (*n* = 40, 30 males, 10 female).

To prevent exposure of the cave environments to *R. rhodochrous* DAP 96253 (RRDAP) volatile organic compounds (VOCs), at each hibernacula bats were exposed to RRDAP VOCs while contained in nylon mesh enclosures placed within 142 liter coolers (Igloo Products Corp, Katy, TX, USA). The non-growth cells of induced RRDAP (Cornelison et al., 2014) were supplied as fermentation cell paste (35 g per cooler) in sealed plastic petri dishes (150 mm × 15 mm; Thermo Fisher Scientific, Waltham, MA, USA). These sealed petri dishes of cell paste were opened and placed on the floor of the treatment cooler without contacting enclosures or bats. An identical opened petri dish was placed in the control cooler without contacting enclosures or bats. Bats were approximately 40 cm from the petri dishes as all bats were observed to be roosting at the top of the mesh containers when placed into the coolers and when the mesh containers were removed. After 48 h of continuous exposure, the bats were moved to the nylon enclosures housed within the PVC frame as described in the “Placement of enclosures” section.

### Mortality tracking

To avoid arousing bats from torpor repeatedly leading to accelerated fat depletion and human-induced death, caves were entered only once, on day 76 of the Breckinridge RRDAP field treatment trial and day 98 of the Rockcastle RRDAP field treatment trial. Caves were entered for routine winter hibernation surveys by the Kentucky Department Fish and Wildlife state biologists. While surveying the rooms where the enclosures had been placed on day 0 of the trials, any bat meeting the criteria for euthanasia (hanging individually near the bottom of the cage and in poor body condition) was euthanized *via* isoflurane overdose. Any deceased bats were removed from the enclosure (only if the surviving bats could remain undisturbed). The identification number was recorded before placing the deceased or euthanized bats into a 50 mL conical centrifuge tube containing 10% buffered formalin (Thermo Fisher Scientific, Waltham, MA, USA).

On day 120 of the Breckinridge RRDAP field treatment trial and day 122 of the Rockcastle RRDAP field treatment trial, the deceased bats were removed from the enclosure and the identification number recorded before placing the bat into a 50 mL conical centrifuge tube containing 10% buffered formalin (Thermo Fisher Scientific, Waltham, MA, USA). Alive bats were examined, identification numbers recorded, and released into the respective hibernacula.

### Gross pathology of deceased bats

At the end of the field treatment trial, all deceased bats underwent gross necropsy to determine the cause of death at the University of Missouri or by the Kentucky Wildlife Veterinarian. We confirmed colonization of the wings by *P. destructans* using Periodic acid-Schiff (PAS) stain (Meteyer et al., 2009), however histopathological analysis of the organs was not pursued due to severe autolysis preventing meaningful histopathological examination.

### Statistical analysis

T-tests were conducted within Excel to ensure there were no significant differences between mass and the estimated percentage of the wings emitting the characteristic orange-yellow fluorescence color between control and treatment groups prior to the start of the trial at each site. This was completed in an effort to reduce potential bias that could result from differences in WNS disease severity. All other analyses were performed in R version 4.2.1 utilizing RStudio version 2022.7.0.548 (R Core Team, 2022; R Studio Team, 2022). Normality was assed using the Shapiro Wilk Test and binary, categorical variables were assessed for tetrachoric correlation using the R-package ‘psych’ (Revelle, 2022). The R-package ‘survival’ (Therneau, 2022) was used to fit the Cox proportional hazard models for each treatment site and to assess overall survival as well as to ensure all Cox model assumptions were met. Interactions between all independent variables were assessed, and non-significant interactions were removed from the models and the analysis rerun without the interaction term to avoid incorrect conclusions (Beck & Bliwise, 2014; Engqvist, 2005). The Cox model tables and survival curves were created using the R-package ‘survminer’ (Kassambara, Kosinski & Biececk, 2021) with multiple records (with consecutive start and end times) for each individual created with bat mortality expressed as a failure event, and surviving bats at each interval were expressed as right-censored data. The R-package ‘countcolors’ was used to determine the percent of the patagia covered by the characteristic fluorescent orange-yellow color indicative of *P. destructans* infection as previously described by the authors (Hooper, Weller & Amelon, 2020).

## Results

### Initial morphometrics and mortality

On the day treatment was initiated, there were no significant differences in mass (Breckinridge *p* = 0.86; Rockcastle *p* = 0.33, overall *p* = 0.36),the estimated percentage of the patagia displaying orange-yellow fluorescent color (Breckinridge *p* = 0.74; Rockcastle *p* = 0.68, overall *p* = 0.66), or the percent of the patagia displaying orange-yellow fluorescent color (Breckenridge, *p* = 0.92, Rockcastle *p* = 0.92, overall *p* = 0.97) as measured by the ‘countcolors’ R-package in the treatment or control groups. On day 0 of the Breckenridge RRDAP VOC field trial, mean fluorescence of the patagia in the control group was 43.0 ± 9.5% (range: 26–57%) and 43.3 ± 11.5% (range: 25–61%) in the treatment group. On day 0 of the Rockcastle RRDAP VOC field trial, mean fluorescence of the patagia in the control group was 41.0 ± 11.9% (range: 19–63%) and 40.7 ± 11.8% (range: 19–75%) in the treatment group. On day 76 and 120 of the Breckinridge RRDAP VOC field treatment trial, 19 and 12 bats remained alive in the treatment group and 19 and 12 remained alive in the control group, respectively. On day 98 and 122 of the Rockcastle RRDAP VOC field treatment trial, 35 and 8 bats in the treatment group remained alive and 34 and 17 bats in the control group remained alive, respectively (Table 1). During the hibernacula surveys and at the end of the study, none of the alive bats met the criteria for euthanasia. On gross necropsy, all deceased bats had completely utilized all fat reserves and the patagiums and uropatagiums had areas with irregular pigmentation and contraction in addition to the loss of tone and elasticity which are all indicative of wing pathology seen in WNS disease progression (Cryan et al., 2010). Histological inspection of the wings revealed *P. destructans* hyphae invasion of the tissue as described by Meteyer et al. (2009), however due to severe autolysis of the majority of bats, histopathology was only used to confirm *P. destructans* infection. These gross and histopathological findings strongly suggest the cause of death for all bats was due to WNS.

**Table 1:** White-nose syndrome treatment trial enrolled, alive bats. The number of *P. destructans* little brown bats enrolled in the study and randomly assigned to the control and treatment groups are shown under the Day 0 column. Hibernacula counts were conducted on day 76 for Breckinridge hibernacula and day 98 for Rockcastle hibernacula with alive bats reported in the respective columns. The survival at the end of the trial is reported for Breckinridge hibernacula on day 120 and day 122 for Rockcastle hibernacula.

Hibernacula	Group	Day 0	Day 76	Day 98	Day 120	Day 122
Breckinridge	Control	20	19	–	12	–
	Treatment	20	19	–	12	–
Rockcastle	Control	40	–	34	–	17
	Treatment	40	–	35	–	8

### Overall survival analysis Cox proportional hazard models

The variables sex and location were strongly correlated (*r*_tet_ = −0.6), and therefore only sex or location was included in each model assessing overall treatment effects. The Cox proportional hazard models assessing the impact of treatment, location, and the interactions on overall survival failed the proportional hazards assumption when location was included (*x*^2^ = 19.09, *p* = 1.2e^−05^). Therefore, stratified cox regression models, with location as a stratified variable, were built to assess the impact of treatment, location, and the interactions. A negative treatment effect trend was found (z-score = 1.60, *p* = 0.10) with no significant interactions (z-score = 0.89, *p* = 0.38).

When treatment, sex, and the interaction of treatment and sex were assessed, the interaction did not significantly alter the risk of death from WNS (z-score = 0.34, *p* = 0.74). After dropping the interaction term to avoid incorrect conclusions (Beck & Bliwise, 2014; Engqvist, 2005), a negative treatment effect trend was found (z-score = 1.81, *p* = 0.07) and males were found to be at significantly lower risk of dying from WNS (z-score = −2.13, *p* = 0.03).

### Breckinridge hibernacula

**Figure 1:**
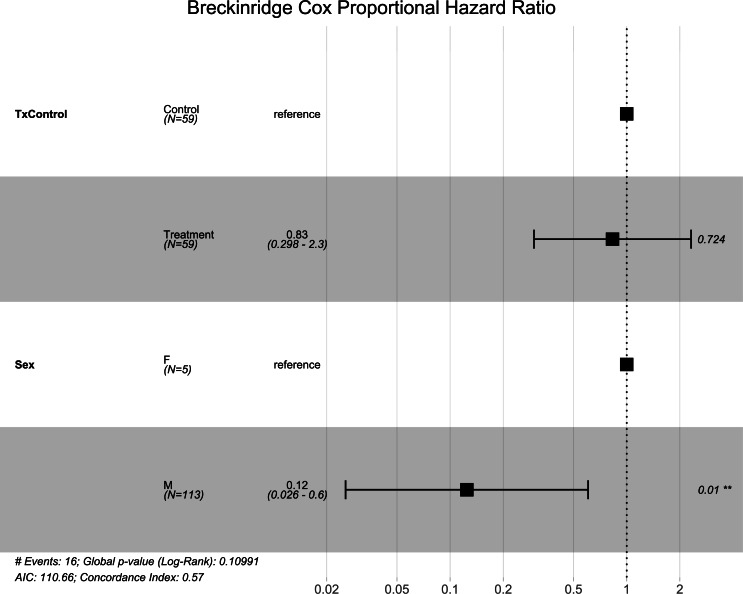
Breckinridge hibernacula Cox hazard proportional model. Visualization of the hazard ratio of all bats enrolled in the Breckinridge treatment trial site. Control bats were the reference population. The degrees of freedom are shown under each categorical variable. The risk ratio (■) and 95% confidence interval are plotted with *p*-values shown to the far right of each set of variables assessed. At Breckinridge hibernaculum, there were no significant differences between the treatment or control groups in cox hazard proportional models (*p* = 0.724); however, male bats had a significantly lower risk of dying from WNS with a hazard ratio of 0.12 (*p* = 0.01) when female bats were the reference population.

**Figure 2:**
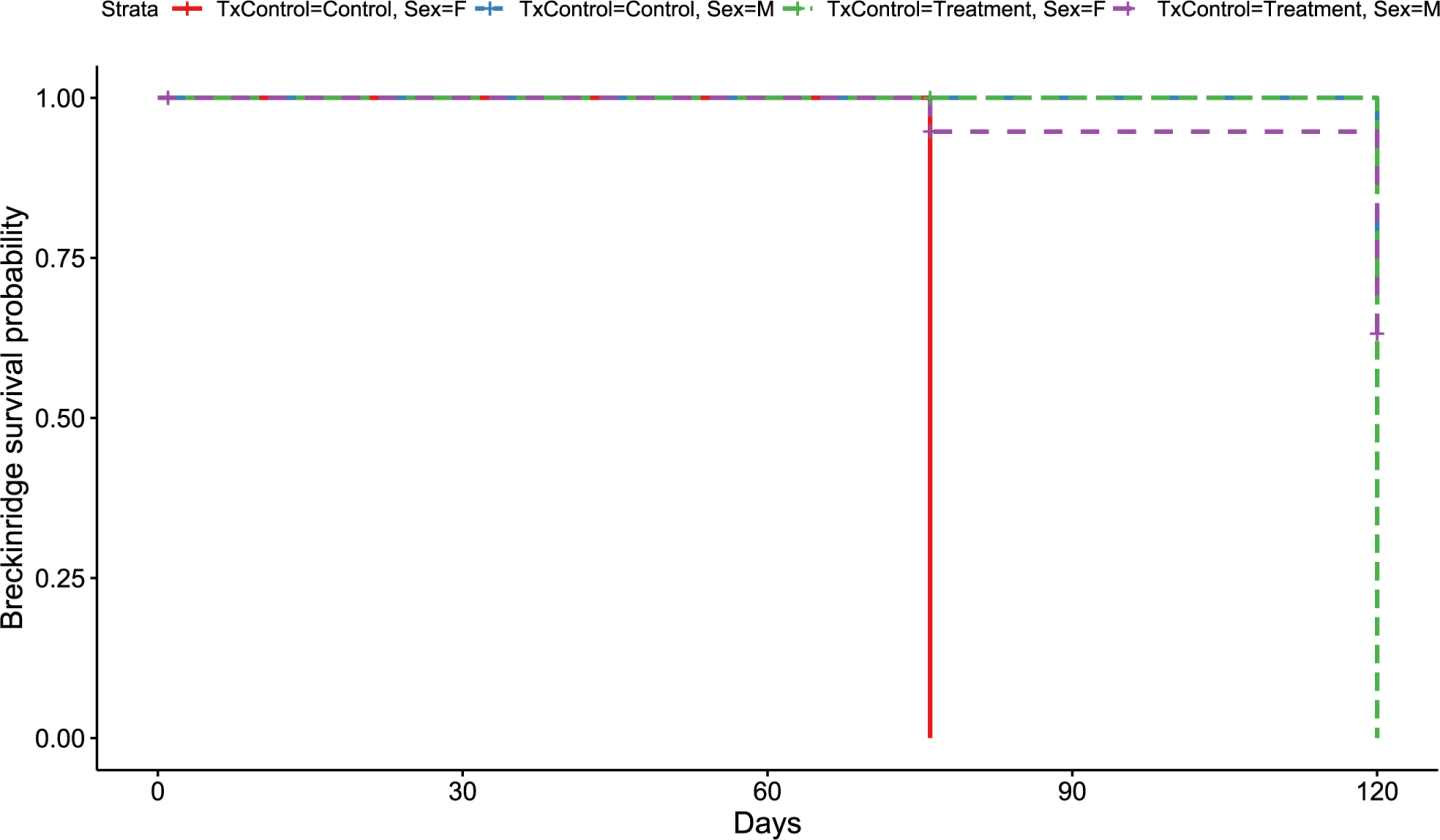
Breckinridge Kaplan–Meier survival curve. Kaplan–Meier survival curve showing survival probably of little brown bats located within the Breckinridge hibernacula treatment trial site based upon the bat’s sex and treatment status. All female control bats (solid red line, *n* = 1), and female RRDAP VOC treatment bats (green dashed line, *n* = 1) died. Survival was 60% for males in both groups (control, blue dashed line, *n* = 12;treatment, purple dashed line, *n* = 12) at the end of the trial.

Similar to the overall models, at the Breckinridge hibernacula, male little brown bats were found to be at significantly lower risk of dying from WNS compared to females (*p* = 0.01, Fig. 1). There were no significant differences found between the control and the RRDAP VOC treatment group (*p* = 0.72, Fig. 1), and both females died in both groups (Fig. 2). Starting mass (z-score −1.07, *p* = 0.28) nor the initial amount of orange-yellow fluorescence as determined by the ‘countcolors’ R-package (z-score = −0.49, *p* = 0.62) impacted the risk of bats dying from WNS.

### Rockcastle hibernacula

**Figure 3:**
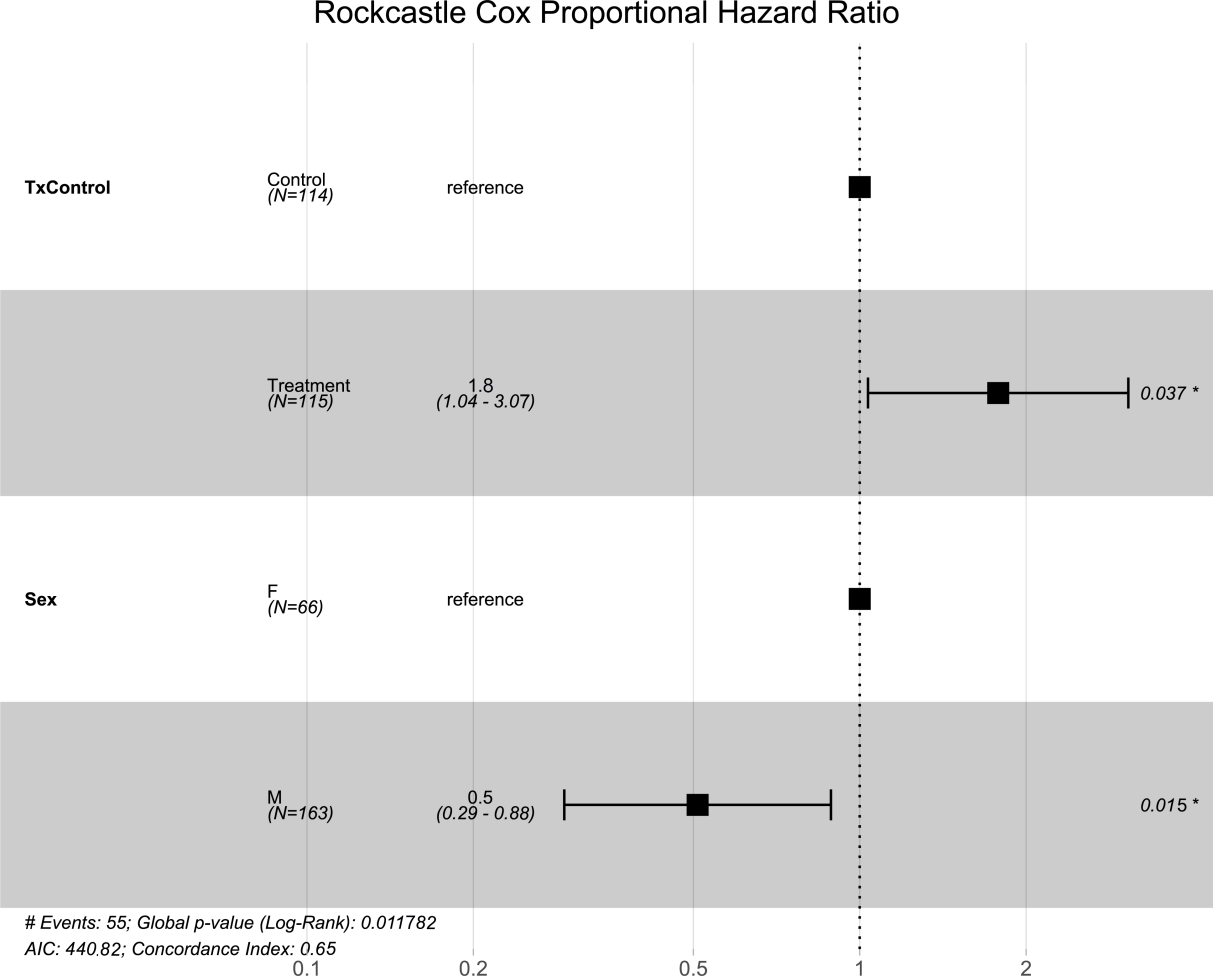
Rockcastle hibernacula Cox hazard proportional model. Visualization of the hazard ratio of all bats enrolled in the Rockcastle treatment trial site. Control bats were the reference population. The degrees of freedom are shown under each categorical variable. The risk ratio (■) and 95% confidence interval are plotted with *p*-values shown to the far right of each set of variables assessed. At Rockcastle hibernaculum, bats in the treatment group were at increased risk of dying from WNS with a hazard ratio of 1.8 (*p* = 0.037). Male bats- had a hazard ratio of 0.5 compared to the reference of female bats, indicating males had a significantly lower risk of dying from WNS (*p* = 0.015).

**Figure 4:**
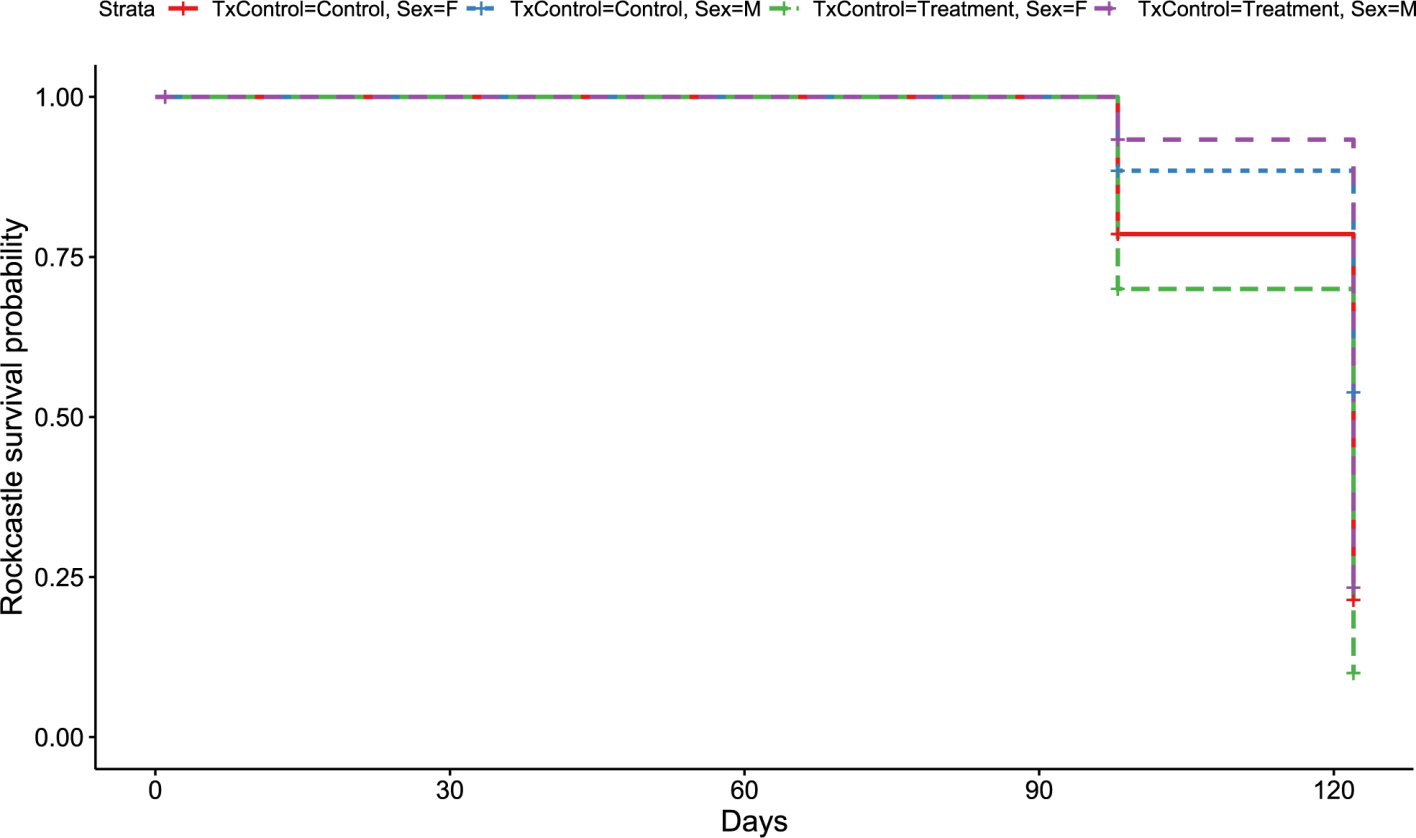
Rockcastle Kaplan–Meier survival curv. Kaplan–Meier survival curve showing survival probably of little brown bats located within the Rockcastle hibernacula treatment trial site based upon the bat’s sex and treatment status. Survival was 20% for those exposed to RRDAP VOCs, with only one female bat (dashed green line) and seven male bats (dashed purple line) alive at the end of the trial. The control group had a survival of 43% with three females (solid red line) and 14 males (dashed blue line) alive at the end of the trial.

Like the overall models, at the Rockcastle hibernacula, male little brown bats were found to be at significantly lower risk of dying from WNS compared to females (*p* = 0.015, Fig. 3). The Cox hazard model revealed bats were at a significantly greater risk of dying from WNS in the treatment group (*p* = 0.037, Fig. 3) and Kaplan Meyer survival curves found significant differences between the control and treatment groups (*p* = 0.024, Fig. 4). Starting mass (z-score = −0.41, *p* = 0.69) nor the initial amount of orange-yellow fluorescence as determined by the ‘countcolors’ R-package (z-score = −0.42, *p* = 0.68) impacted the risk of bats dying from WNS.

## Discussion

Previous studies assessing VOCs produced by induced RRDAP cells and RRDAP non-growth fermentation cell paste yielded complete inhibition of *P. destructans* for more than 80 days *in vitro* and these VOCs were also documented to completely inhibit *P. destructans* from colonizing bat wing explants (Cornelison et al., 2014). With these promising results further supported by 60% survival of bats exposed to RRDAP VOCs compared to 0% survival in the control group in a small laboratory trial (Pierce et al., 2017), a two-site *in situ* treatment trial was initiated with a slightly longer exposure time of 48 h. Our initial *ex situ* trial documented after 24 h of RRDAP VOC exposure, the survival of *P. destructans* infected bats was significantly increased (Pierce et al., 2017). Additionally, it was not feasible to start our treatment trials on the same day as the sites were approximately 4 h driving distance apart and it took an entire day to start the trial at one site. Being unable to start both trials on the same day, we could start them subsequently; and then return to the first site to remove the RRDAP non-growth fermentation cell paste without extending the time beyond 72 h, or the maximum amount of time little brown bats had been exposed to the RRDAP VOCs without observed ill effects. With the selected 48 h exposure time, the increased mortality associated with RRDAP VOC exposure *in situ* was unexpected. Also surprising was the increased mortality observed in the little brown bats treated with RRDAP VOCs only occurred at one of the hibernacula sites, the Rockcastle county location.

While the Cox hazard models for both field sites revealed male bats were at a lower risk of dying, this should be interpreted with caution due to the unequal and low numbers of females. Only 2 of the 40 bats in the Breckridge trial and 24 of the 80 bats at the Rockcastle trail were female. Ideally, an equal number of males and females both within and between treatment and control groups should have been included at both sites. However, since fewer females than males were captured at both sites, this supports previous reports that males are more likely to survive WNS (Grieneisen et al., 2015) as well as supports the outcomes of the hazard models. Our results suggest that future WNS treatment trial studies should aim to include equal numbers of males and females to assess potential sex-effects of the treatments as sex may affect survival.

Two hibernacula sites were selected to avoid pseudoreplication, however pseudoreplication at each hibernacula site was unavoidable as all bats were housed together. Individualized housing would have prevented the little brown bats from exhibiting normal clustering behavior which is thought to be essential for surviving hibernation by reducing metabolic requirements for euthermy after torpor arousal (Boyles & Brack, 2009; Czenze, Park & Willis, 2013) and reducing evaporative water loss (Thomas & Cloutier, 1992). Therefore, bats were not forced to roost individually in order to avoid increased physiological and behavior stress.

Cages for the treatment trial were placed in the exact location where bats were hibernating before being captured to allow bats to continue hibernating as close to their selected microenvironment as feasibly possible. This was deliberately pursued to help mitigate the additional stress associated with a caged experimental *in situ* field trial design. Additionally, we employed the identical nylon caging system *in situ* as was used in the laboratory trials previously conducted by the authors. Overwinter survivorship was 100% for healthy little brown bats housed in these cages and no abnormal behaviors were observed (Pierce et al., 2017) indicating the cage size and material was appropriate for this species.

However, it is important to recognize a caged field study has limitations. For instance, there may be increased stress from altered behavior (*e.g.*, bats may be disturbed when other bats arouse from hibernation and they are unable to select or move to different hibernation roosting locations; Hoyt et al., 2019; Turner et al., 2015). To date, all WNS studies assessing potential mitigations strategies *in vivo* have been solely caged-based (Kilpatrick et al., 2016; Overton et al., 2016; US Fish and Wildlife Service, 2022a; Vonhof et al., 2018; Vonhof et al., 2017) or include at least one group within cages (Hoyt et al., 2019). The survival outcome of each individual can be known in caged treatment trials since bats are unable to leave the hibernacula or be consumed by predators when cages are designed appropriately. Despite the limitations of a caged-based experimental design, because the control and treatment groups were identically housed, any negative impacts, including increased risk of death, should have equally exerted on both groups thereby mitigating the potential for bias in our survival analysis. This is supported by our observation of equal mortality in all groups at the Breckinridge hibernacula (Fig. 2).

The Breckinridge hibernacula survival rates were overall higher at 60% for both the control and RRDAP VOC exposed bats compared to the 20% survival of the RRDAP VOC exposed bats and the 43% survival rate of the control bats at the Rockcastle hibernacula. The higher mortality observed in both the treatment and control groups at Rockcastle, could possibly be explained by the temporal disease dynamics of WNS. The mortality rate for hibernating little brown bats is recognized to be the highest during the second and third year of infection and by the fourth year begins to level out (Langwig et al., 2012). Rockcastle hibernacula was in the third year post-WNS detection and suffered from 80% mortality in the RRDAP VOC exposed bats and 57% mortality in the controls bats whereas the 40% mortality at Breckinridge hibernacula could be due to this site being in its fourth year post-WNS detection. This temporal dynamic likely also explains why fewer bats were able to be enrolled into the treatment trial at Breckinridge as the overall population of hibernating little brown bats was half the size of the Rockcastle population.

It is unclear if the temporal dynamics could have played a role in the observed mortality differences between treatment and control groups at Rockcastle. Due to clinical signs of WNS being found at all hibernacula initially evaluated for inclusion in this study, we were unable to find healthy bats, unaffected by WNS, to inoculate with a known amount of *P. destructans* conidia. As a result, we selected bats with natural, active *P. destructans* infection. Unable to perform qPCR in the field the day of the trial to determine fungal load, we opted to screen bats using long-wave UV light (Turner et al., 2014) in an effort to balance groups for fungal loads. McGuire et al. documented that UV fluorescence detection is 100% specific with 73% sensitivity with 0 false positives reported in the study (McGuire et al., 2016). Furthermore, it has been shown fungal load to be correlated with UV fluorescence (McGuire et al., 2016) which supports our use of long wave UV light for estimating initial fungal load and disease severity. Therefore, it is unlikely that disease severity bias at the start of our experiment led to the higher mortality observed in the RRDAP VOC treatment group at the Rockcastle site.

*Pseudogymnoascus. destructans* fungal load has been shown to alter the bacterial microflora on little brown bat wings (Lemieux-Labonté et al., 2017). While multiple *R. rhodochrous* strains have been identified as normal microflora on the skin of North American bats (Hamm et al., 2017; Lemieux-Labonté et al., 2017), *Rhodococcus* species are found to be more abundant on the skin of WNS positive little brown bats. It is unclear why *Rhodoccocus* species are significant indicators of WNS positive hibernacula (Lemieux-Labonté et al., 2017). Though several of these strains (*e.g.*, *R. rhodochrous* (AC 241)) have been shown to completely inhibit the growth of *P. destructans* (Hamm et al., 2017) similar to the RRDAP VOCs employed in this study. Contrary to this, some species in the *Rhodoccocus* genus have been found to actually increase the *P. destructans* fungal load based upon quantitative PCR (Grisnik et al., 2020). Due to the limitations on not being able to handle the bats the day of death to collect wing swabs, we were unable to measure *P. destructans* loads on the deceased bats nor monitor the microbiota. Therefore, it is unclear if the higher mortality at Rockcastle was caused by an increase of *P. destructans* growth or if the RRDAP VOCs exposure negatively altered the microflora on the wing membranes, leading to increased mortality. Additionally, while the 360–385 nm UV wavelengths used in this study do not significantly impact the survival of *P. destrucans* (Palmer et al., 2018), it is unclear if the UV exposure during photographing of the UV transilluminated wings altered the wing microflora.

RRDAP VOCs were selected over a probiotic application of RRDAP to the wing membrane of the bats because VOC exposure can occur without any handling of bats—if VOC distributor systems are installed where bats typically hibernate before winter hibernation. Additionally, these distributor systems would allow periodic VOC exposure. The goal of periodic exposure would be to increase the overwinter survival of the most severely affected species. It is important to recognize this treatment was designed to mitigate but not cure the disease. The hibernacula environment serves as an abiotic reservoir of *P. destructans* (Hoyt et al., 2015b; Lorch et al., 2013), and if fungal loads can be reduced leading to decreased overwinter mortality, affected hibernating bat species may have adequate time to develop resistance or tolerance mechanisms against WNS such as been suggested to have occurred in European bat species (Bandouchova et al., 2015; Puechmaille et al., 2011). Although it has been suggested that resistance may be developing in some persisting North American bat populations (Langwig et al., 2017), it is unclear if these small surviving populations would meet the minimum viable population size required for the species survival (Rai, 2006; Reed et al., 2003), therefore there is continued interest in pursuing disease mitigation strategies, including treatments, for WNS.

## Conclusions

When treating little brown bats *in situ* with RRDAP VOCs, lower survival was observed in one treatment group while concurrently the second treatment group showed no increase or decrease in survival rates when compared to the control groups. While RRDAP VOC exposure was shown to effectively inhibit *P. destructans in vivo*, it may increase the risk of death from WNS *in situ*; therefore, there is not enough evidence to support conducting large scale exposure of hibernating bats to RRDAP VOCs as part of WNS disease mitigation strategies.

##  Supplemental Information

10.7717/peerj.15782/supp-1 File S1 Supplemental Figures

## Additional Information and Declarations

### Field Study Permissions

The following information was supplied relating to field study approvals (*i.e.*, approving body and any reference numbers): Authorized state biologists employed by the Kentucky Department of Fish and Wildlife were permitted for all described collection, treatment, and sampling procedures (KDFWR-W-2014-01).

### Data Availability

The following information was supplied regarding data availability: The R code and raw data are available at GitHub and Zenodo: https://github.com/MicroBatVet/RRDAP_WNSFieldTrial
MicroBatVet. (2023). MicroBatVet/RRDAP_WNSFieldTrial: v1.1 release (v1.1). Zenodo. https://doi.org/10.5281/zenodo.8084459
