# Supplementary material for: Contact-independent exposure to Rhodococcus rhodochrous DAP96253 volatiles does not improve the survival rate of Myotis lucifugus (little brown bats) affected by White-nose Syndrome
Source: PeerJ. 2023 Oct 18;11:e15782. doi: 10.7717/peerj.15782 (PMC10590100; doi:10.7717/peerj.15782)

Contact-independent exposure to *Rhodococcus rhodochrous* DAP96253 volatiles does not improve the survival rate of *Myotis lucifugus* (little brown bats) affected by White-nose Syndrome

Sarah E. Hooper^1,2*^ and Sybill K. Amelon^3*^

^1^ Department of Biomedical Sciences, Ross University School of Veterinary Medicine, Basseterre, St. Kitts and Nevis, West Indies

^2^ Department of Veterinary Pathobiology, College of Veterinary Medicine, University of Missouri, Columbia, Missouri, United States of America

^3^ United States Department of Agriculture United States Forest Service, Northern Research Station, Columbia, Missouri, United States of America

Supporting Information:

**S1. Images of suspended nylon enclosure within wire mesh enclosure**


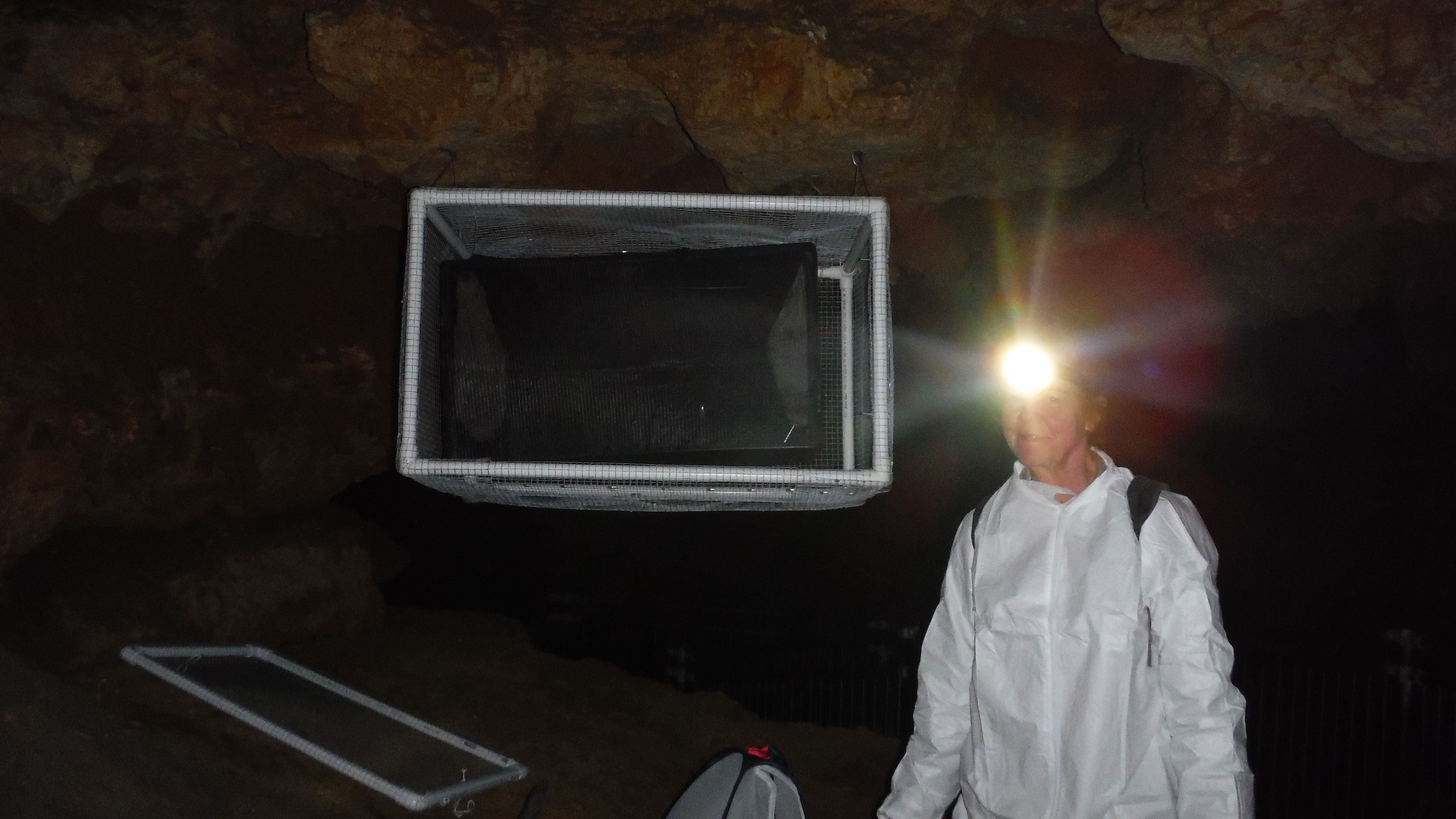

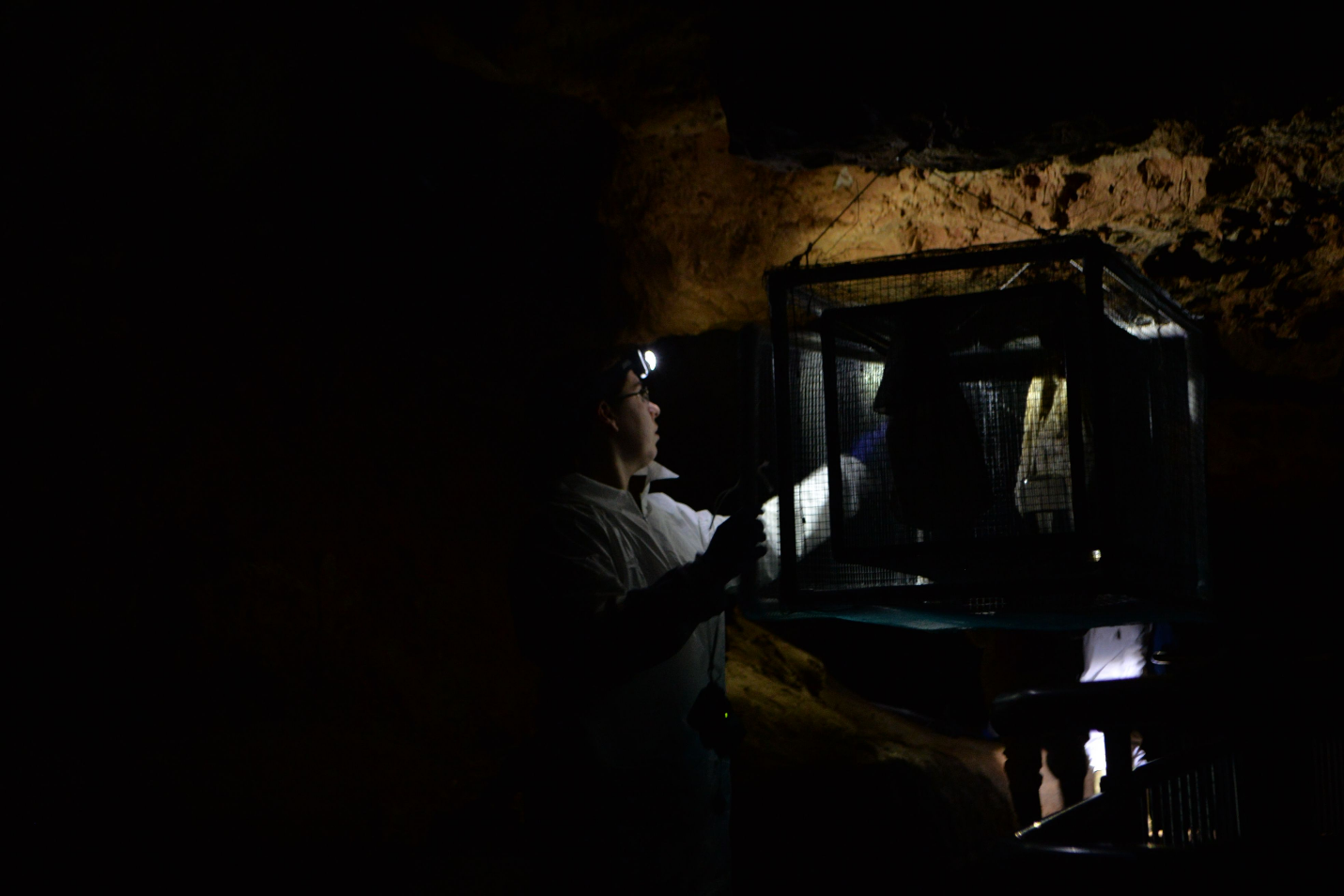

Supplement: File S1 [file peerj-11-15782-s001.docx]
